# Telemedicine, Telehealth and m-Health: New Frontiers in Medical Practice

**DOI:** 10.3390/healthcare2020250

**Published:** 2014-06-13

**Authors:** Elizabeth A. Krupinski, Ronald S. Weinstein

**Affiliations:** 1 Department of Medical Imaging & Arizona Telemedicine Program, University of Arizona, 1609 N Warren Bldg 211, Tucson, AZ 85724, USA; 2 Arizona Telemedicine Program & Southwest Telehealth Resource Center, University of Arizona Health Sciences Center, P.O. Box 245105, Tucson, AZ 85724, USA; E-Mail: rweinstein@telemedicine.arizona.edu

Telemedicine is changing the practice of medicine. It is part of the ever-growing use of communications technology in health care being used in prevention, disease management, home health care, long-term (chronic) care, emergency medicine, remote medical imaging, and many other applications. The pace at which telemedicine is being adopted and integrated into the healthcare enterprise is exponential and, for many (even those in the field!), it is often difficult to keep up with all of the changes occurring. Thus, it is useful periodically to stand back and summarize recent advances, to take stock, analyze where we have been, and project where we are headed.

This Special Issue of *Healthcare* features invited papers by experts, on important topics in the fields of telemedicine, telehealth and mHealth (*i.e.*, mobile Health). After a gestation period of a half a century, since the first multi-specialty telemedicine service was created in Boston at the Massachusetts General Hospital in the late 1960s, telemedicine is now reaching its stride as an important and practical way to deliver a broad spectrum of healthcare services to patients. Over 60 subspecialties of medicine and nursing are involved with telemedicine and telehealth. For example, teleradiology has become a standard-of-care for night time coverage in rural and urban hospitals. Telestroke networks may become the next essential “urgent care” service. Telepsychiatry is commonplace in many practice environments. The “smart phone” is being shown to be well suited for use in many mobile telehealth applications. Today, thousands of healthcare apps are being developed, tested and marketed to healthcare providers as well as patients. This Special Issue of *Healthcare* features original papers by leaders in telemedicine whose work is at the leading edge of this rapidly advancing field.

The issue features a paper by Tim Hunter, MD on teleradiology that will prove very useful to those “shopping” for a teleradiology service entitled “University-Based Teleradiology in the United States” [1]. It reviews the long-standing and very successful University of Arizona’s teleradiology practice, as well as university-based teleradiology practice in the U.S. in general. It very nicely describes the unique benefits and challenges of engaging a university-based radiology practice for teleradiology services, including a discussion of reimbursement models, consultation models, and what types of questions one should ask when searching for a teleradiology service. Readers will find Table 4 in the article very useful as it succinctly summarizes nine key recommendations for a successful university-based teleradiology practice that both those seeking and those providing services can readily refer to.

Dr. Elizabeth Krupinski also submitted a paper, “Human Factors and Human-Computer Considerations in Teleradiology and Telepathology”, that deals with teleradiology, but from a very different perspective that also addresses telepathology [2]. Both of the specialties are in many ways more developed than other clinical specialties, perhaps because both are traditionally image based and for the most part have fewer direct patient encounters. This paper reviews the rather unique digital viewing environments for teleradiology and telepathology, providing a new look at how to optimize case reading environments and address human factors issues. It reviews key components that need to be optimized for effective and efficient practice of teleradiology and telepathology using conventional digital imaging workstations, as well as some of the newer mobile viewing applications.

Although the practice of telemedicine is slowing being integrated into traditional healthcare delivery systems, in many ways it still requires a dedicated workspace or at least special attention to converting an existing workspace into one that is amenable to the tele-encounter. In “Telemedicine Workplace Environments: Designing for Success”, Dr. Krupinski further explores the topic of ergonomics in telemedicine by looking at the workspace environment [3]. Although the future practice of telemedicine is likely to be more of a mobile-based practice and centered more in the home than it is now, it is still very important to consider ways to optimize the design of clinic-based telemedicine facilities. This is true on both ends of a consultation—where the patient is and where the consultant is. The paper covers everything from lighting and acoustical considerations to what color the walls should be painted, outlining a variety of easy to take steps to optimize rooms for telemedicine.

Setting up the basic technology and networking infrastructure for telemedicine is one thing, but convincing practitioners to actually use telemedicine is often another challenge unto itself. There are many ways to champion telemedicine and find converts, but giving someone a practice guideline developed by experts engaging telemedicine on a daily basis can often be the most convincing and useful tool you can provide. In “Standards and Guidelines in Telemedicine and Telehealth”, Dr. Krupinski provides an overview of the development of guidelines and standards that help insure effective and safe delivery of quality healthcare [4]. The paper reviews critically important, sustained, efforts by the American Telemedicine Association in developing telemedicine practice guidelines, review the role of research in guidelines development, review data regarding their use, and discuss some of the additional areas where guidelines are still needed. There are lots of references to the existing guidelines that readers can download freely and adopt today!

Finally, there is a paper by Chan *et al*., “Mobile Tele-Mental Health: Increasing Applications and a Move to Hybrid Models of Care” that very nicely describes the vast potential of mobile devices for psychotherapy, psychosocial interventions, and other behavioral health programs [5]. Particular emphasis is placed on novel interventions that integrate sensors to monitor patients and algorithms that provide “interpretations” of these data to help predict when patients require some sort of intervention. As with the first article on teleradiology, the benefits and challenges associated with mobile devices are reviewed and a number of recent key studies in the topic summarized. Some key clinical scenarios in which mobile options are proving to be useful are described, followed by a discussion of what is still needed to make these devices are part of regular care, including more research validating them, more clinical trials, and adoption of existing guidelines.

The final few sentences of the Chan article provide a glimpse of what is certainly going to be healthcare in the very near future—a “hybrid” practice in which mobile and other emerging technologies are used regularly as part of every practice to effectively and efficiently monitor, connect with, educate and care for patients. Each of the articles in this Special Issue will provide readers with insights into the practice of telemedicine that will hopefully inspire them to join the telemedicine community sooner rather than later as it clearly is here to stay!
